# Olfactory receptor genes cooperate with protocadherin genes in human extreme obesity

**DOI:** 10.1007/s12263-015-0465-3

**Published:** 2015-05-06

**Authors:** Edwin C. M. Mariman, Radek Szklarczyk, Freek G. Bouwman, Erik E. J. G. Aller, Marleen A. van Baak, Ping Wang

**Affiliations:** Department of Human Biology, NUTRIM School for Nutrition, Toxicology and Metabolism, Maastricht University Medical Centre, Maastricht, The Netherlands; Department of Clinical Genomics, Maastricht University Medical Centre, Maastricht, The Netherlands; Laboratory of Biochemical Genetics, Department of Clinical Genetics, University Hospital Maastricht, Maastricht University Medical Centre, Maastricht, The Netherlands

**Keywords:** Extreme obesity, Genetic predisposition, Olfactory system, Protocadherins

## Abstract

Worldwide, the incidence of obesity has increased dramatically over the past decades. More knowledge about the complex etiology of obesity is needed in order to find additional approaches for treatment and prevention. Investigating the exome sequencing data of 30 extremely obese subjects (BMI 45–65 kg/m^2^) shows that predicted damaging missense variants in olfactory receptor genes on chromosome 1q and rare predicted damaging variants in the protocadherin (PCDH) beta-cluster genes on chromosome 5q31, reported in our previous work, co-localize in subjects with extreme obesity. This implies a synergistic effect between genetic variation in these gene clusters in the predisposition to extreme obesity. Evidence for a general involvement of the olfactory transduction pathway on itself could not be found. Bioinformatic analysis indicates a specific involvement of the PCDH beta-cluster genes in controlling tissue development. Further mechanistic insight needs to await the identification of the ligands of the 1q olfactory receptors. Eventually, this may provide the possibility to manipulate food flavor in a way to reduce the risk of overeating and of extreme obesity in genetically predisposed subjects.

**Electronic supplementary material:**

The online version of this article (doi:10.1007/s12263-015-0465-3) contains supplementary material, which is available to authorized users.

## Introduction

Worldwide, the incidence of obesity has increased dramatically over the past decades and all kinds of measures ranging from dietary and lifestyle interventions to bariatric surgery are being taken to revert this situation, albeit with limited success. Apparently, more knowledge about the complex etiology of obesity is needed in order to find additional approaches for treatment and prevention. Obesity and parameters of adiposity are to a considerable extent determined by genetic factors. Heritability estimates for obesity in different studies amount to at least 0.4 (Walley et al. 2006; Farooqi and O’Rahilly 2007; Berndt et al. 2013). Knowledge of those factors and the processes that they influence provides possibilities for diagnostic testing and counseling in addition to development of novel intervention methods. Unfortunately, more than 90 % of the genetic background is still unexplained (Speliotes et al. 2010; Loos 2012; Wheeler et al. 2013); thus, a further search for obesity-related genes is warranted. Recently, using functional clustering analysis of genes with rare variants, we have shown that in people with extreme obesity (BMI 45–65 kg/m^2^), the protocadherin (PCDH) genes on chromosome 5q31 harbor a surplus of rare variation with a moderate-to-high predicted biological effect (Mariman et al. 2014).

Studies in the mouse have provided evidence for a prominent role of the PCDH gene cluster in the development of the olfactory system (Hasegawa et al. 2012; Ledderose et al. 2013; Hirano et al. 2012). We therefore wondered whether the previously identified rare PCDH gene variation (Mariman et al. 2014) and damaging variation in the genes of the olfactory transduction pathway might work synergistically in the predisposition to extreme obesity. In that case, damaging missense variants in the genes of the olfactory transduction pathway might occur more often in the extreme obese subjects carrying the rare potentially damaging variation in the PCDH genes than in the non-carrier subjects. In the present study, we analyzed genetic variants of the olfactory system in relation to the earlier reported variants in the PCDH genes in 30 extreme obese subjects.

## Materials and methods

### Subjects

The selection of the subjects has been described before (Mariman et al. 2014). In short, from 561 obese individuals who attended the private obesity clinic CO-EUR (http://www.co-eur.eu/), 30 (19 females, 11 males) relatively young (average age 29.7 years, range 19–40.4 years) extremely obese (average BMI 51.1 kg/m^2^, range 45.3–65.1 kg/m^2^) Caucasian subjects were selected. Written informed consent for genetic studies was obtained from all participants, and permission was granted by the Medical Research Ethics Committee of the Maastricht University Medical Center. For these subjects, only weight, length, age and sex were registered, and no clinical biochemical data were available.

### DNA isolation and sequencing

 From peripheral blood leukocytes, genomic DNA was isolated using the QIAamp DNA blood kit (Qiagen, Amsterdam, The Netherlands) and was then outsourced for whole exome sequencing in a CLIA-certified laboratory (EdgeBio, http://www.edgebio.com/). The Nimblegen capture kit was used for the selection of genomic coding regions, followed by sequencing on the Illumina HiSeq 2000.

For our exome sequencing, the target 64 Mb consisted of 34-Mb exons. The average median whole targeted sequencing read depth was 50. For all samples, 94.6 % of bases had a read depth >10, and 87.2 % of bases had a read depth >20. 84–86 % of the reads mapped to the target regions, and 14–16 % was off target.

Sequence results were compared with GRCh37 (b37) as the reference genome (http://www.ncbi.nlm.nih.gov/projects/genome/assembly/grc/human/). For every subject, data were returned in excel-readable format as a SNP file and an indel file (insertion/deletion). Only the SNP files were used for the present study. Information was provided concerning genetic data (chromosome, cytoband, reference position, gene, zygosity, rs number, alleles, etc.), quality of the sequencing (Phred quality score, depth) and predicted biological effect by various methods (SNPeffect (Reumers et al. 2005), SIFT score (http://sift.jcvi.org/), PolyPhen2 class and score (http://genetics.bwh.harvard.edu/pph2/), etc.). In addition, the corresponding KEGG pathway identifier(s) (http://www.genome.jp/kegg/) were given for the genes.

### Identification of PCDH-variant carriers

Previously, we analyzed the sequencing data for the presence of rare variants with a moderate-to-high predicted biological damaging effect in functional gene clusters (Mariman et al. 2014). PCDH gene clusters on chromosome 5q31 displayed a high frequency of such variants in the extreme obese cohort. In the present study, we compared genetic data between carriers of these PCDH variants and the individuals not carrying such variants in our cohort (non-carriers). In Table 1, PCDH-variant carriers are listed with information of the PCDH genes in which their rare variants occurred.

**Table 1:** Subjects carrying rare variants with a predicted moderate-to-high biological effect in PCDH genes

PCDH gene	Subject code											
	3	8	13	14	16	19	21	22	23	26	28	30
Alpha-cluster												
PCDHA6				X								
PCDHA8									X			
PCDHA12							X					
Beta-cluster												
PCDHB2										X		
PCDHB3			X									
PCDHB4												X
PCDHB8						X						
PCDHB10				X								
PCDHB14				X								
PCDHB16				X								
Gamma-cluster												
PCDHGA8					X			X				
PCDHGB1						X					X	
PCDHGB7	X	X										

### Selection of variants

**Fig. 1:**
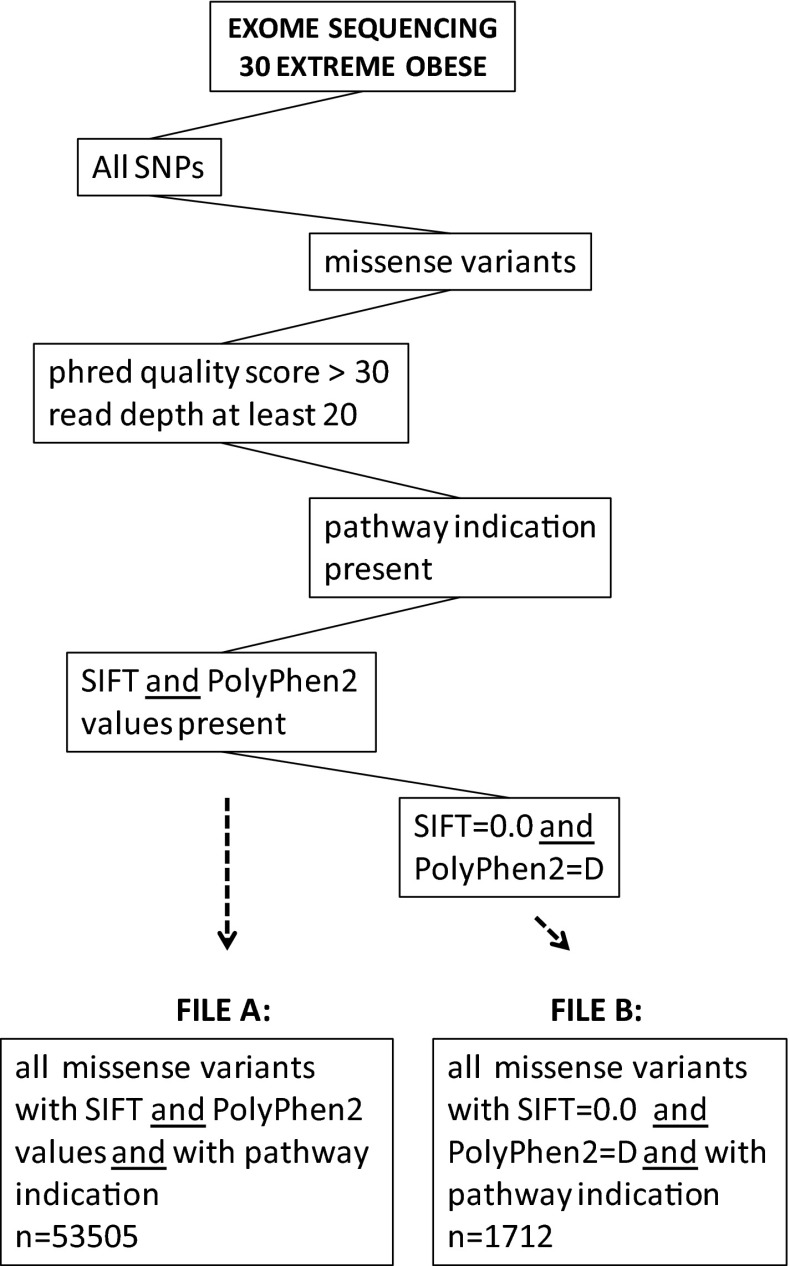
Selection scheme for obtaining data files

For the present study, we focused on missense variants with a predicted damaging impact on protein function present in genes that had already been functionally annotated in KEGG pathways. The selection route for these variants is depicted in Fig. 1. In short, all missense variants detected in the 30 extremely obese subjects with a Phred quality score >30 (equivalent to a base call accuracy >99.9 %) and a read depth of at least 20, with KEGG pathway ID(s) and with scores on biological impact of the variant allele were selected. From the resulting list, a subset of predicted damaging variants was further selected based on the presence of both a SIFT score of 0.0 and a PolyPhen2 class ‘D’. The SIFT score is based on the premise that the amino acid order of a protein is related to evolution. A SIFT score below 0.05 means that the amino acid is highly conserved and substitution may result in damaging of the function of the protein. In this regard, a SIFT score of 0.0 indicates a high probability of damaging (Ng and Henikoff 2003). PolyPhen2 takes structural features of the protein into account to classify amino acid substitutions like occurrence in a functional module and/or impact on the 3D structure (Adzhubei et al. 2010). Substitutions are quantitatively scored, but also qualitatively classified as ‘benign’ (B), ‘possibly damaging’ (P) and ‘probably damaging’ (D). We took the last category into account.

Thus, two SNP data files were composed: file A: all annotated missense variants and file B: predicted damaging missense variants. The amount of damaging missense variants were evaluated by two parameters: (1) the ratio of damaging missense variants to total missense variants, which equals the number of missense variants per pathway in file B/number of missense variants of the same pathway in file A and (2) the burden, which equals the sum of damaging missense variants per pathway per person.

### Computational assessment of the function of PCDH gene clusters

The genes of the PCDH alpha-, beta- and gamma-clusters were used to infer coexpression data. Publically available gene expression data (GEO http://www.ncbi.nlm.nih.gov/geo/) for human and mouse were used to find genes that coexpress with PCDH clusters. The approach is an extension of the one published by Baughman et al. (2009). Shortly, using an in-house computational pipeline, all microarray experiment consisting of multiple expression measurements was evaluated separately. Evaluation consists of calculating the average Pearson’s coexpression of each gene of the three PCDH clusters. If the coexpression of the system (e.g., alpha-cluster) is congruent (on average, genes are up- and downregulated together), a high weight is assigned to the experiment. Analogously, if genes within the system do not appear to coexpress with each other in the experiment, the experiment itself receives a low weight. After assigning weights to experiments, for each experiment, the coexpression of each human gene is calculated with each of the clusters as average Pearson’s correlation of expression. The results of coexpression with the clusters are then integrated for all experiments (in a weight-sensitive manner, as previously calculated) and for both human and mouse. This gives the final list of genes coexpressing with each cluster for which the top 100 was selected as genes most likely functionally related to PCDH gene sets. As can be expected, those PCDH cluster-linked gene sets contained several PCDH genes. For further analysis, those genes were removed resulting in a set of 83 genes for the alpha-cluster, 70 genes for the beta-cluster and 65 genes for the gamma-cluster.

Next, these gene sets were analyzed by the program DAVID (http://david.abcc.ncifcrf.gov/) to find functional aspects that are specific for the different gene sets, which would serve as an indicator for the function of the PCDH cluster genes. Particular analyses involved the beta-linked gene set against all genes in three sets as the background, beta-linked gene set against all human genes, and alpha- and beta-linked gene sets against all human genes.

### The Genome of the Netherlands as control

We used the general Dutch population data as the control. The Genome of the Netherlands database is represented by the general Dutch population (www.nlgenome.nl) (Consortium 2014). That project covered an area of 41,543 km^2^ with more than 17 million inhabitants and included the province Limburg from where the present cohort was assembled. The number of subjects is 498 in the extractable genome database. A set of 353 non-pseudo-genes of olfactory receptors (OR) was used to extract genome information. When no information of the variant was available in the Genome of the Netherlands, we used the data from large populations in the dbSNP database (www.ncbi.nlm.nih.gov/snp).

### Statistics

From the 30 extreme obese subjects, 12 are PCDH-variant carriers, whereas 18 are non-carriers (Table 1). Chi-square analysis was used to test whether the total number of damaging missense variant hits in OR genes or in subsets of those genes was different between the two groups. A hit is defined as a hetero- or homozygous carrier of the altered allele. A similar analysis was done to investigate the hits of damaging missense variants in OR genes between carriers of the previously identified PCDH alpha-, beta- or gamma-cluster variants and the rest of the subjects. Mann–Whitney test was used as an alternative method to compare PCDH-variant carriers with non-carriers. A *P* = 0.05 was regarded as the threshold for significant deviation from the null hypothesis.

Genetic association was assessed by Chi-square analysis comparing the observed genotype frequencies in this extreme obese cohort with the genotype frequencies reported in the Genome of The Netherlands database or reported in the dbSNP database.

The comparison on the burden of damaging missense alleles was analyzed by Chi-square with Yates’ correction. In total, 2266 missense polymorphisms and 319 damaging missense polymorphisms were detected either in the present cohort or in the controls. Those were used to extract the number of total missense and damaging missense alleles as the input for the data shown in Table 3.

## Results

### Relation between the PCDH cluster and the olfactory transduction pathway

**Fig. 2:**
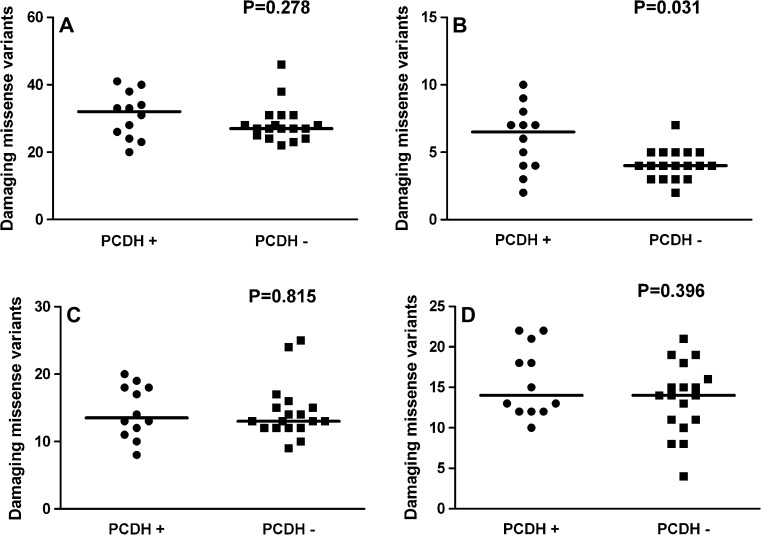
Relation between olfactory transduction gene variants and PCDH gene variants. The distribution of predicted damaging missense variants in genes of the olfactory transduction pathway (hits per person) is shown in subjects with (PCDH+) and without (PCDH−) previously identified rare PCDH variants. **a** All variants annotated to olfactory transduction genes; **b** variants in olfactory transduction genes on chromosome 1; **c** variants in olfactory transduction genes on chromosome 11; **d** variants in olfactory transduction genes all other chromosomes than 1 and 11. The *P* value above each graph was of Mann–Whitney test

In the olfactory transduction pathway, we scored in total 883 hits (hetero- or homozygous carrier of the altered allele) of predicted damaging missense variants in 110 polymorphisms. All genes appeared to belong to the superfamily of OR genes (Malnic et al. 2004). Almost half of these hits, 432, were in genes on chromosome 11 and 146 in genes on chromosome 1, whereas 307 were in genes distributed over 15 other chromosomes. For the total set of hits of predicted damaging missense variants in the olfactory transduction pathway, and in the three subgroups according to the chromosome location, we tested by *χ*^2^ analysis whether there might be a higher number of OR-variant hits in PCDH-variant carriers than in non-carriers. The results showed that significance was only reached for the chromosome 1 subgroup (*P* = 0.023). Mann–Whitney test gave similar results with significance only for the chromosome 1 subgroup (*P* = 0.031, Fig. 2b).

To check the specificity of such co-damaging missense variation between two pathways, we tested the tight junction and ABC receptor pathways in a similar way. These two pathways were selected due to their high ratio of damaging variants as mentioned later. For both pathways, no significant relation between their variants and PCDH variation was detected by *χ*^2^ analysis (*P* = 0.78 and *P* = 0.16, respectively) nor by Mann–Whitney test (*P* = 0.79 and *P* = 0.06, respectively). Because of the relatively low number of variants in these two pathways, no subdivision according to chromosomes or genetic loci was made.

### Detailed analysis of olfactory transduction genes on chromosome 1q

The 146 hits of predicted damaging missense variants in OR genes on chromosome 1 were all located on the long arm and divided over two loci at 1q23 (*n* = 26) and 1q44 (*n* = 120). In order to assess whether the observed relation with PCDH variation could be ascribed to one of those loci or to both, *χ*^2^ analysis and the Mann–Whitney test were used to compare the hits of variation between PCDH-variant carriers and non-carriers. *χ*^2^ analysis resulted in a trend for difference for 1q23 (*P* = 0.062) and 1q44 (*P* = 0.092), whereas the Mann–Whitney test resulted in significance for 1q23 (*P* = 0.037) and a trend for 1q44 (*P* = 0.060). Apparently, both loci contribute to the effect suggesting that genes from both locations cooperate with PCDH genes in the genetic predisposition to extreme obesity.

Table 2 gives a detailed overview of the involved OR genes together with the polymorphisms and the IDs of the subjects who are carriers of the risk alleles. It shows that relatively common polymorphisms as well as rare variants are involved. For all except one variant, the observed allele frequencies were in line with that of the control general populations. A comparison of the observed genotype distribution of the G/T variant in the OR14C36 gene (rs28545014; D231Y) with that reported for the Genome of the Netherlands indicated a genetic association between this missense variant and extreme obesity (*P* < 0.0001). Using frequencies of the CS-Agilent population or of the ESP-cohort populations from dbSNP did not alter this finding (*P* = 0.001).

**Table 2:** Detailed overview of OR genes with damaging missense variants on chromosome 1q

Subject code	Locus	Gene symbol	DNA polymorphism	rs number	Amino acid polymorphism	Observed allele frequency (alleles)	Allele frequency in GON or dbSNP
*28*	1q23	OR10J1	T/C	rs35634161	M70T	0.983/0.017 (59/1)	0.981/0.019^c^
*13*		OR10K2	T/G	rs79869455	L39R	0.983/0.017 (59/1)	0.990/0.010^b^
11, 12, *13*, *14*, *16*, *19*, *21*, *28*, *30*		OR10T2	G/A	rs61818749	C89Y	0.850/0.150 (51/9)	0.909/0.091^c^
2, 4, 7, *8*, 9, 11, *13*, *14*, 17, 18, *23*, *26*, 27, *30*		OR10Z1	A/C	rs857685	N294T	0.733/0.267 (44/16)	0.764/0.236^c^
1, 2, *3*, 5, 6, 9, 10, 11, 12, *13*, *14*, 15, *16*, 17, 18, *19*, 20, *21*, *22*, *23*, 24, 25, *26*, 27, *28*, 29, *30*	1q44	OR2AK2	G/A	rs4478844	V203 M	0.367/0.633 (22/38)	0.384/0.616^c^
1, 4, 5, 6, 7, 9, 12, *13*, 15, *16*, 17, *19*, *21*, *23*, 24, *26*, 29		OR2G2	T/C	rs10925085	L167P	0.667/0.333 (40/20)	0.597/0.403^c^
*26*		OR2G3	C/A		A237D	0.983/0.017 (59/1)	
*13*		OR2M3	T/A	rs144171858	I289 N	0.983/0.017 (59/1)	0.998/0.002^a^
1, 24		OR2M5	C/A	rs41304157	A237D	0.967/0.033 (58/2)	0.981/0.019^c^
*30*		OR2T11	T/G	rs75010552	M203R	0.967/0.033 (58/2)	0.992/0.008^c^
*13*		OR2T2	G/A	rs145665149	G234D	0.983/0.017 (59/1)	0.999/0.001^b^
*13*		OR2T3	G/C	rs148805039	G21A	0.983/0.017 (59/1)	0.998/0.002^a^
*3*, 4, 6, 7, *8*, 9, 10, 11, *13*, 17, *19*, *21*, 25, *26*, *28*, 29, *30*			G/A	rs148025314	C132Y	0.817/0.183 (43/17)	0.811/0.189^a^
*8*, *19*, 25			T/C	rs148748995	F183S	0.950/0.050 (57/3)	0.997/0.003^a^
1, 4, 12, *13*, *14*, 17, *19*, 20, *21*, 25, *26*, *28*, 29		OR2T6	T/G	rs954475	S243A	0.767/0.233 (46/14)	0.852/0.148^c^
*3*, 4, *8*, *30*		OR2T34	G/A	rs147489167	C132Y	0.917/0.083 (55/5)	0.993/0.007^a^
2, 4, 5, *8*, *13*, *14*, *19*, 25, *26*, *30*			T/C	rs150608839	F183S	0.834/0.166 (50/10)	0.896/0.104^a^
2, 5, 6, 7, *8*, 10, *14*, 15, *16*, 18, *26*, 27, *28*, *30*		OR14C36	G/T	rs28545014	D231Y^##^	0.700/0.300 (42/18)	0.431/0.569^c^
*3*, 4, 6, *8*, 10, 11, *22*, *30*		OR14I1	A/G	rs55871516	Y216C	0.867/0.133 (52/8)	0.868/0.132^c^

Carriership of missense variants with a predicted damaging effect in olfactory receptor genes on chromosome 1q is shown together with their observed and reported allele frequency

Subject code in italics: PCDH-variant carriers; in roman: non-carriers

^##^Genetic association with extreme obesity (*P* < 0.0001)

^a^CS-Agilent, ^b^ ESP-cohort populations; ^c^ Genome of the Netherlands (GON)

### Detailed analysis of 1q OR genes and the PCDH clusters

**Fig. 3:**
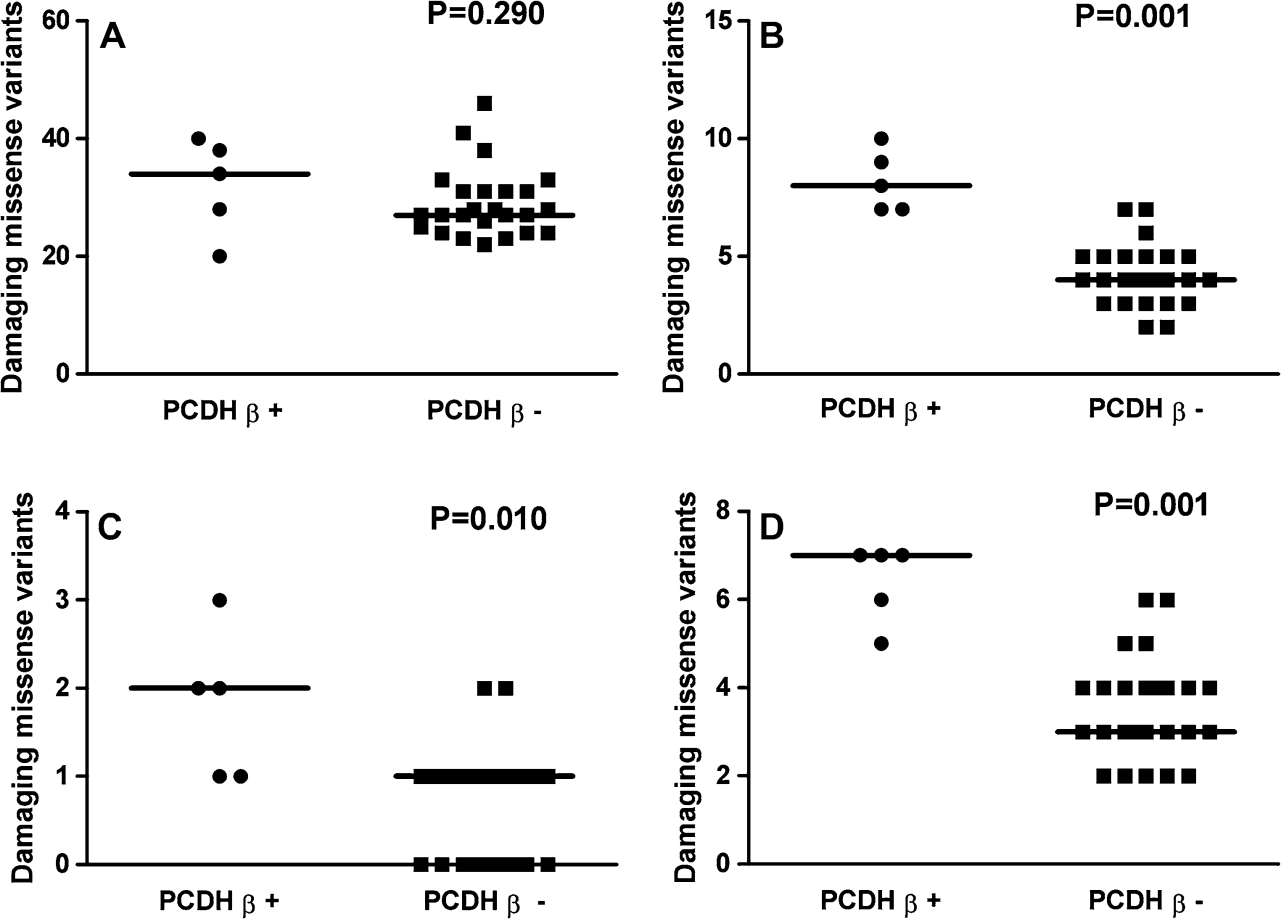
Relation between OR gene variants and PCDH beta-cluster gene variants. The distribution of predicted damaging missense variants in OR genes (hits per person) is shown in subjects with (PCDH β+) and without (PCDH β−) previously identified rare variants in the PCDH beta-cluster genes. **a** All variants annotated to olfactory transduction genes; **b** variants in OR genes on chromosome 1; **c** variants in OR genes at 1q23; **d** variants in OR genes at 1q44. The *P* value above each graph was of Mann–Whitney test

To determine whether the variation in the 1q OR genes was linked with one of the PCDH clusters, the occurrence of hits of 1q OR predicted damaging missense variants was compared per cluster among the subjects with and without a PCDH variant. Similar analyses were performed also for the 1q23 and 1q44 loci separately. Subjects (ID # 14 and #19) with variants in genes of two different PCDH clusters were counted in both of the clusters. Significance for *χ*^2^ analysis was only observed for subjects with a PCDH beta-cluster variant regarding all 1q OR variants (*P* < 0.0001, Fig. 3b), variants on 1q23 (*P* = 0.014, Fig. 3c) and on 1q44 (*P* = 0.004, Fig. 3d). Mann–Whitney test gave similar *P* values. It indicates that the genetic cooperation between damaging variation in the OR genes on chromosome 1q and variation in the PCDH genes is confined to the PCDH beta-cluster genes and does not occur with the alpha- and gamma-clusters.

### Extended functional assessment of PCDH gene clusters

 The functions of PCDH gene clusters were indirectly assessed by the functions of their linked genes. Sets of coexpressing genes were generated for each of the PCDH clusters (Table S1) and functionally annotated to obtain an indication of the specific function of the different PCDH gene clusters. The outcome (Table S2) indicates that the PCDH beta-cluster genes are linked to the control of tissue development (‘DNA binding,’ ‘transcription regulation,’ ‘cell cycle,’ ‘cell cycle phase’), whereas the PCDH-alpha and gamma genes seem to be more engaged in neuron organization and function (‘neuron projection,’ ‘cytoplasm,’ ‘synapse,’ ‘axon’).

### The relevance of high number of damaging missense variants in the olfactory transduction pathway in extreme obesity

Olfactory transduction pathways showed high number of damaging missense variants in our extreme obesity cohort. We evaluated the ratio of predicted damaging missense variant hits to total missense variant hits for various pathways. The olfactory transduction pathway together with the tight junction pathway and ABC transporters pathway formed the top three (Table S3). However, comparing our cohort with the control on the number of damaging missense alleles in the olfactory transduction pathway per person showed that extreme obese subjects even had a lower burden than the general population (Table 3).

**Table 3:** Comparison of the burden for the olfactory transduction pathway

	Extreme obesity	Genome of the Netherlands	*P* value
Total missense alleles per person	599	447	<0.0001
Ratio of damaging alleles to total missense alleles	5.8 %	8.9 %	<0.0001
Burden of damaging missense alleles per person	34.5	39.8	<0.0001

## Discussion

The main olfactory system consists of the olfactory bulb, where at the glomeruli neuronal cells are connected with OR cells. When volatile chemicals are recognized by ORs, a signal is transduced into perception and generates the sensation of smell (Cleland 2014). Obviously, smell is an important determinant of food selection and consumption (Jaeger et al. 2013; Blundell and Finlayson 2004; Shepherd 2006; Reed and Knaapila 2010; Sorensen et al. 2003). Close to 400 human OR genes distributed over 21 human chromosomes constitute one of the largest gene families (Hasin-Brumshtein et al. 2009; Malnic et al. 2004). The high redundancy of ORs may make the system more ‘tolerant’ to damaging mutations. The embedded high genetic variability of OR genes is well recognized (Mainland et al. 2014).

In the present study, we also confirmed that a relatively large amount of missense variants with a predicted damaging impact on protein function occurred in extreme obesity. However, compared to the general population, it even seems that extreme obese subjects carried less damaging alleles. Nevertheless, different number of subjects per sequenced cohort and different methods used in exome capture and sequencing may eventually lead to the difference. In the general Dutch population, only 233 out of total 319 damaging missense polymorphisms were detected. Because low-frequent damaging variants in the general population are easy to be missed in our small cohort, this might lead to an under-estimated burden in our extreme obesity cohort in comparison with that of the control. A rough calculation taking this under-estimation into account would lead to no significant difference on the burden. For the common 86 damaging missense polymorphisms, there was also no difference on the burden between the extreme obese and general Dutch population (29.1 vs. 29.5). These results suggest at least that the high damaging genetic variation of the olfactory transduction pathway, in either ratio or absolute burden, is not specific to extreme obesity. However, as indicated in the present and in few other studies, there can still be genetic association between particular ORs and obesity (Choquette et al. 2012; Jaeger et al. 2013; Lunde et al. 2012; Jarick et al. 2011).

Here, we have shown that common and rare damaging missense variants in OR genes on chromosome 1q are carried more often by extreme obese subjects who also carry variants with a moderate-to-low predicted biological effect in the genes of the PCDH cluster on chromosome 5q31, in particular in the genes of the beta-cluster. Our observations suggest a synergistic effect of genetic variation in genes of the PCDH beta-cluster and of the olfactory transduction pathway on chromosome 1. The fact that the rare variants in the PCDH genes were previously shown to be implicated in extreme obesity [8] suggests that variation in the OR genes and PCDH genes exerts an additive effect on the risk of extreme obesity.

The link between the 1q OR and PCDH beta-cluster genes suggests that they are involved in the same biological process. The PCDH genes play a role in establishing neuronal connectivity and synaptic specificity and maturation in various regions of the central nervous system (Li et al. 2010). Although no clear specific functions have been annotated on the PCDH clusters, some experimental evidence has shown non-exclusive roles of PCDH clusters in the development of the olfactory system. During development of the mouse, the maturation of the olfactory system requires the coalescence of homotypic olfactory sensory neurons into glomeruli of the olfactory bulb, which are specified by ORs. Hasegawa et al. (2012) have proposed that this is accomplished by two counteracting processes, of which the coalescence is led by axon guidance molecules like ephrin, semaphorin, plexin, BIG-2 and kirrel2/3, and the repulsion of homotypic axons is mediated by the alpha-protocadherins. However, the development of the olfactory system is more complex and is also accompanied by the expression of the PCDH beta- and gamma-cluster genes (Hirano et al. 2012; Ledderose et al. 2013). The beta-cluster has been proposed to play an important role in neuron cell individuality [10]. It is in line with our bioinformatics analysis that suggests a specific role of those genes in the control of gene expression and cell division, and as such in tissue development. Our results also suggest that the PCDH beta-cluster genes are involved in the development of glomeruli of the olfactory system that in humans are specified by the OR genes on chromosome 1q.

While studies on gene function in the mouse indicate that the PCDH and OR genes mediate the development and fine-tuning of the olfactory sensory system, our results suggest that in humans OR genes on chromosome 1q and PCDH beta-cluster genes may form two parts of one axis. The co-impairment of these two parts can contribute to the risk of extreme obesity at a relatively young age. Indeed, the human olfactory system and its connection with the brain are complex, and its importance for food selection and food intake is obvious but has been largely overlooked (Shepherd 2006; Palouzier-Paulignan et al. 2012). As a consequence, it is unknown how the co-impairment of OR genes and PCDH genes influences human olfaction. Theoretically, a poor performance of this axis may inhibit the maturation of certain olfactory glomeruli and induce less strong synaptic connections with certain sensory neurons leading to impairment of particular sense signals (Reed and Knaapila 2010), which may reduce the food-rewarding circuits and stimulate the body to eat more for compensation. On the other hand, the inhibition of certain types of olfactory glomeruli may lead to oversensitivity of others triggering stronger hedonic signals to the brain (Blundell and Finlayson 2004). In this sense, altered olfaction may also stimulate the food attraction. Unfortunately, in humans, only a few ORs have been annotated for their ligands. The gene OR10J5 located at 1q23 is the human homolog of the murine gene for OR23, which recognizes the odorant lyral with a perception of lemony odor (Malnic et al. 2004). The OR2M7 gene on 1q44 is likely to be involved in the ability to smell methanethiol, which is set free in the urine after eating asparagus (Eriksson et al. 2010). Obviously, knowledge about the ligands of the specific ORs on chromosome 1q seems crucial for solving the puzzle of these genes and their role in food intake.

The number of genetic studies looking into inter-individual variation in smell perception is now rapidly increasing (Keller et al. 2007; Reed and Knaapila 2010). However, studies making the link with food intake are still very limited. Lunde et al. (2012) showed that genetic variation in the OR7D4 gene determines the level of aversion to consume pork from male pigs containing androstenone. Moreover, genetic variation in this gene and other OR7 genes was reported to display genetic association with eating behavior and adiposity (Choquette et al. 2012). In another study, Jaeger et al. (2013) were able to link preference for foods and products with added beta-ionone to genetic variation in the OR5A1 gene. Interestingly, a common copy number variant region was identified on chromosome 11q11 associated with obesity and exclusively covering three OR genes, OR4p4, OR4S2 and OR4C6 (Jarick et al. 2011). Our present results may add to those studies by suggesting genetic association between variation in the OR14C36 gene on 1q44 and extreme obesity. Altogether, it is surprising how little genetic evidence has been reported for the involvement of the OR genes in weight regulation. This may be due to the fact that complex gene–gene interactions play an important role instead of single variations in the OR genes. In this respect, a polygenic model will be more suitable to analyze OR gene effects for obesity just like we have shown here.

It has been shown that morbidly obese subjects have an olfactory dysfunction in comparison with moderately obese individuals (Richardson et al. 2004). This has led to the debate whether weight gain leads to impairment of the olfactory function or whether it is the other way round. The fact that weight loss after a gastric bypass does not improve the olfactory sensation suggests that it is the olfactory dysfunction that contributes to extreme obesity (Richardson et al. 2012). Our results may support the concept that weight gain can be a consequence of an impaired olfactory system, since a genetic influence from the olfactory system together with PCDH genes underlying the risk of extreme obesity is suggested here.

Recently, it was shown that ORs are expressed in other tissues than the olfactory bulb. After feeding obesity-prone and obesity-resistant rats a high-fat diet for 14 days, microarray analysis showed up-regulation of four OR genes in duodenal enterocytes of the obesity-prone animals (Primeaux et al. 2013). It suggests that these receptors play a role in sensing nutrients or their metabolites as they pass through the lumen of the gut, and assist in regulating their uptake. In lung epithelium of the mouse, it was found that beta-carotene simultaneously up-regulates 26 OR genes and 9 genes of the PCDH beta-cluster. It was proposed that those genes might be active in the highly innervated pulmonary neuroendocrine cell clusters (van Helden et al. 2010), which may have various functions like lung tissue repair, sensing volatile chemicals and monitoring oxygen levels in the inhaled air (Song et al. 2012; Gu et al. 2014). A link with chronic obstructive pulmonary disease (COPD) has been suggested (Gu et al. 2014), but not with obesity. Obviously, our knowledge about the functional cooperation of PCDH and OR genes in the human body is still very limited, and this cooperation may not only occur in the extreme obesity context. Until we obtain more knowledge, our present findings cannot be definitely ascribed to aspects of smell sensation.

The present findings are based on the analysis of whole exome sequencing data and partly comply with the suggested guidelines for quality (MacArthur et al. 2014). For instance, we predicted missense variants to be damaging by using a combination of two functional annotation programs, SIFT and PolyPhen2. Also, we did not assume that the implicated variants or combinations thereof were fully penetrant. Nevertheless, our work is based on a limited number of subjects and our findings need to be confirmed in a similar, independent study.

## Conclusions

In summary, we found that predicted damaging missense variants in OR genes on chromosome 1q and rare damaging variants in PCDH beta-cluster genes on 5q31 co-localize in subjects with extreme obesity, suggesting a synergistic effect in the predisposition to this condition. Further, mechanistic insight may come from the identification of the ligands of the 1q ORs linked to the PCDH beta-cluster. Eventually, this may provide the possibility to manipulate food flavor in a way to reduce the risk of overeating and extreme obesity in some genetically predisposed subjects.

## Electronic supplementary material

Table S1 List of genes coexpressed with PCDH gene clusters. Microarray analysis results stored in public databases were analyzed for genes showing coexpression with the different PCDH gene clusters (XLSX 13 kb)

Table S2 Functional annotation analysis of genes coexpressed with PCDH gene clusters. The genes that coexpress with PCDH gene clusters were analyzed with regard to annotated function using DAVID (XLSX 21 kb)

Table S3 Total and damaging missense variation in pathways. The pathways that had at least 500 hits for total missense variants and at least a 1 % ratio of damaging missense variant hits to total missense variant hits are listed (DOCX 16 kb)
