# Supplementary material for: Olfactory receptor genes cooperate with protocadherin genes in human extreme obesity
Source: Genes Nutr. 2015 May 6;10(4):16. doi: 10.1007/s12263-015-0465-3 (PMC4420755; doi:10.1007/s12263-015-0465-3)
Supplement: Supplementary file 3 — Total and damaging missense variation in pathways. The pathways that had at least 500 hits for total missense variants and at least a 1 % ratio of damaging missense variant hits to total missense variant hits are listed (DOCX 16 kb) [file 12263_2015_465_MOESM3_ESM.docx]

**Supplement Table S3. Total and damaging missense variation in pathways.**

| **Pathway** | **KEGG pathway ID** | **Total missense** | **Predicted damaging** | **Ratio^*^** |
| --- | --- | --- | --- | --- |
|  |  | **Variant hits** | **missense variant hits** | **(%)** |
| **tight junction** | hsa04530 | 739 | 106 | 14.3 |
| **olfactory transduction** | hsa04740 | 13552 | 883 | 6.5 |
| **ABC transporters** | hsa02010 | 1171 | 67 | 5.7 |
| **peroxisome** | hsa04146 | 520 | 18 | 3.5 |
| **MAPK signaling pathway** | hsa04010 | 1040 | 31 | 3.0 |
| **calcium signaling pathway** | hsa04020 | 850 | 24 | 2.8 |
| **steroid hormone biosynthesis** | hsa00140 | 611 | 17 | 2.8 |
| **glycolysis/gluconeogenesis** | hsa00010 | 615 | 16 | 2.6 |
| **pentose and glucuronate interconversions** | hsa00040 | 552 | 13 | 2.4 |
| **complement and coagulation cascades** | hsa04610 | 1088 | 24 | 2.2 |
| **insulin signaling pathway** | hsa04910 | 563 | 12 | 2.1 |
| **regulation of actin cytoskeleton** | hsa04810 | 1400 | 28 | 2.0 |
| **glycerophospholipid metabolism** | hsa00564 | 559 | 10 | 1.8 |
| **neuroactive ligand-receptor interaction** | hsa04080 | 1678 | 30 | 1.8 |
| **PPAR signaling pathway** | hsa03320 | 761 | 11 | 1.4 |
| **purine metabolism** | hsa00230 | 921 | 12 | 1.3 |
| **ubiquitine mediated proteolysis** | hsa04120 | 559 | 7 | 1.3 |
| **chemokine signaling pathway** | hsa04062 | 723 | 9 | 1.2 |
| **Fc gamma R-mediated phagocytosis** | hsa04666 | 580 | 7 | 1.2 |
| **pathways in cancer** | hsa05200 | 2270 | 27 | 1.2 |
| **focal adhesion** | hsa04510 | 2924 | 31 | 1.1 |
| **ECM-receptor interaction** | hsa04512 | 2655 | 27 | 1.0 |

*The ratio of missense variant hits with a predicted damaging effect to total number of missense variant hits per pathway.
